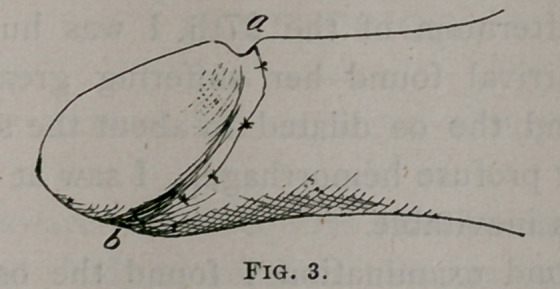# Note on the Operation of Circumcision

**Published:** 1893-10

**Authors:** N. Davies-Colley


### NOTE ON THE OPERATION OF CIRCUMCISION.

BY N. DAVIES-COLLEY.

The operation of circumcision is so simple that the most meagre details with respect to its performance have been given by the writers of our surgical text-books It is, however, an operation very frequently performed by the most inexperienced members of our profession; and a great deal of discomfort, and even serious injury, may result from certain mistakes, against which, as it seems to me, our surgical writers have not sufficiently warned their readers.

Mr. Howse, in a previous number of our Reports,* has clearly pointed out one evil result which may follow the usual practice of slitting up the mucous membrane of the prepuce along the dorsum of the glans penis, and turning the two halves outwards and downwards, so that the redundant tissue forms a knob beneath the glans, which, in some cases, is almost as big as the glans itself. But there are other evil results which he has not mentioned.

Thus, occasionally, we find that so much skin has been removed that during erection the integuments of the organ are very tightly stretched. · One cause of this result is the direction given in one of our text-books that, as the first step of the operation, a dressing forceps should be applied to the penis at the level of the corona glandis. The prepuce is then drawn forwards, with the closed blades of the forceps grasping the skin, while the glans is allowed to slip backwards. If, as not unfrequently occurs, the glans penis has already, from the effects of cold, shrunk back into the skin near the root of the penis, the shave of the knife in front of the forceps will take away nearly all the skin of the organ. Another, and perhaps more serious error, is to fail in dividing the mucous membrane of the prepuce as far back as the sulcus behind the glans, and then to leave so much of this mucous membrane behind that it

* ser. iii, vol. xviii.

closes up again, so as to produce a still greater constriction in front of the glans than before the operation. I once had a child brought to me who had to pass his urine with great difficulty through an orifice which would hardly admit the point of a pin. He had been circumcised a few months previously, and the operator attributed this obstruction to the neglect of the child's nurse, who had not attended to his injunction that the foreskin should be drawn back every day. The injunction would not have been needed if a sufficient amount of the mucous membrane had been removed in the first operation. Another, but less serious result of operating in the ordinary fashion is that a triangular surface is often left beneath the glans without any skin to cover it; and the recovery of the patient is retarded while the surface heals over by granulations.

The method I am about to describe is one which I have used for more than fifteen years. I have never seen any description of a similar method, but I cannot say how far the plan is original with myself. It is possible that for some part of it I may be indebted to the late Mr. Cock; but I do not remember to have seen him perform the operation of circumcision, nor do I know of any description of his method of operation.

The patient being under an anæsthetic, an assistant should grasp the foreskin somewhat obliquely with a pair of dressing forceps forwards and downwards, taking care that the glans penis is not included. He should first, however, observe that the penis is not unduly retracted, and the incision in the skin should begin upon the dorsum at a point corresponding to that part of the glans which is half-way between the meatus urinarius and the corona. If the penis is retracted he must apply the forceps further forwards. Before closing the forceps, care must of course be taken that the glans penis has been allowed to slip back behind the blades, and that only the foreskin is grasped. The surgeon should now apply the knife in front of the forceps, and cut through the dorsal half of that which is grasped. Next, after removing the forceps, he should cut through the rest of the skin with a pair of scissors downwards and forwards, so as to leave a sharp point in the middle of the under surface of the penis (*vide* figures 1 and 2.) (The object of this pointed projection is to fill up subsequently the triangu-

FIG. 1.

lar interval which is otherwise left when that portion of the mucous membrane of the prepuce, to which the frænum is attached, is removed.)

FIG. 2.

The second stage of the operation is to slit up the mucous lining of the prepuce which still envelops the glans penis. This should be done as usual, along the dorsal surface, either with the blunt-pointed scissors, or with a director and knife. The former is preferable, unless the tissues are so thick as to make it uncertain whether one blade of the scissors has entered the urethra. All adhesions should be torn through with the nail or a probe, until the corona and the sulcus behind it have been fully exposed. The surgeon should then clip away with the scissors all the mucous membrane of the prepuce except a frill, so to speak, a sixth of an inch broad all the way round the corona from the dorsal aspect to the frænum. Usually but little hæmorrhage occurs ; but what there is can readily be stopped by twisting the bleeding points with a Spencer Wells' forceps, or by tying a fine catgut ligature around it. The margin of the skin will now be found to fit accurately along the cut edge of the mucous membrane, and it should be attached by fine sutures of silk or catgut, which should be passed by means of an ordinary sewing needle, close to the edges of the skin and mucous membrane, and drawn tightly, so that they do not require to be subsequently removed. The wound should now be sprinkled with iodoform powder, and covered with a film of absorbent cotton-wool, which should be painted over with flexible collodion. This dressing will usually remain on until the wound is healed; but if, on account of the restlessness of the patient, it becomes loose or drops off, a little boracic ointment should be smeared on in its place.

The advantages I claim for this mode of operation are: (1) there is no possibility for recurrence of the phimosis, as the mucous membrane, to which the constriction is due, has been completely taken away; (2) there is never any lump formed behind the frænum by the infiltration of reflected mucous

FIG. 3.

membrane; (3) the skin is left in such a quantity as to allow erection of the organ to take place without any tension; and (4) the whole of the wound usually heals up by primary union.

In fig. 1 I have indicated the line of incision, *a b*, through the skin, and at *c* the stricture of the mucous membrane, which is the cause of the phimosis; the finer dotted line represents the mucous membrane lining the prepuce and covering the glans penis.

In fig. 2 is shown the adjustment of the pointed process of skin (*b*) in the angle left by the remains of the frænum. The dotted line (*d e*) indicates the edge left to the skin on the under side of the penis by the usual mode of operation, and the triangle (*b d e*) the bare surface which has to heal by granulation.

Fig. 3 represents the penis after suturing the edge of skin (*a b*) to the strip of mucous membrane which is left along the back and sides of the corona.—*Guy's Hos, Rep. vol. xlix.* 1892.

## Figures and Tables

**Fig. 1. f1:**
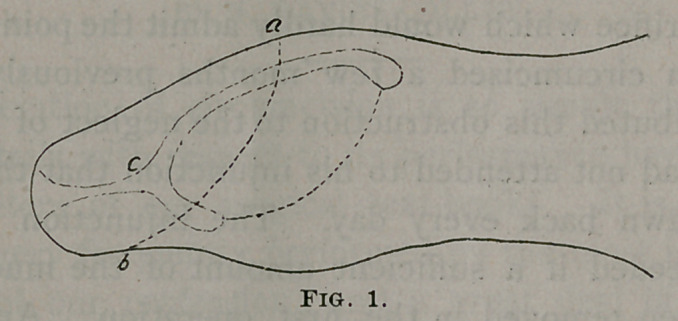


**Fig. 2. f2:**
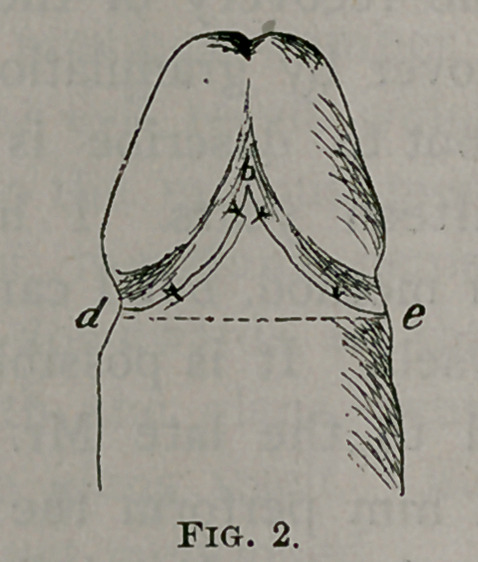


**Fig. 3. f3:**